# Perioperative Mortality Rate in a Low-Resource Non-governmental Organisation Setting

**DOI:** 10.7759/cureus.79439

**Published:** 2025-02-22

**Authors:** Yuki Julius Ng, Ravindranath Shroff

**Affiliations:** 1 General Surgery, International Medical University, Kuala Lumpur, MYS; 2 General Surgery, Vivekananda Memorial Hospital, Sargur, IND

**Keywords:** general surgery, global surgery, low-resource setting, non-governmental organization, perioperative mortality

## Abstract

Introduction

A significant portion of the global population lacks access to safe and affordable surgical and anaesthesia care, with the majority of those affected residing in low- and middle-income countries. As a low-middle-income country and one of the most populated nations, India faces substantial challenges in addressing this healthcare gap. We aimed to descriptively assess the perioperative mortality rate (POMR) of a low-resource non-governmental organisation hospital in India and assess the preparedness of surgical services.

Methodology

We performed a retrospective study by collecting the surgical volume from the operation theatre registry. All recorded deaths about surgery during the timeframe of data collection and investigated each death. We have also assessed the hospital’s preparedness for surgical services.

Results

The operation theatre registry recorded 1,860 patients over five years who underwent major operations. The perioperative mortality was three (0.16%). The case mix done under obstetrics and gynaecology was 1,046 (56.2%), general surgery at 614 (33.0%), and orthopaedics at 200 (10.8%). After adjustment, we found that our average surgical volume per year was 448 (95% CI: 391.52-504.96). Emergency surgeries had a relative risk of 1.9 (95% CI: 0.172-20.87) higher than elective surgeries. Our situational analysis of surgical preparedness shows that Vivekananda Memorial Hospital (VMH) is a well-prepared hospital to provide 32 out of 42 essential surgeries, as described by the Disease Control Priorities. This hospital fulfilled the infrastructure and equipment required for an ideal 100-bed hospital, except for having a blood bank.

Conclusion

In a low-resource non-governmental organisation setting such as VMH, POMR can meet global standards. Surgical care is accessible and affordable to the poorest of the population.

## Introduction

Two-thirds of the global population do not have timely access to safe and affordable surgical and anaesthesia care, and 90% of the low-middle income countries (LMICs) do not have access to both. An estimated 313 million surgeries are done every year, but only 6.5% of those surgeries are done in the LMICs [1]. The Lancet Commission on Global Surgery (LCoGS) provided six indicators to measure access to surgical care. Perioperative mortality rate (POMR) was indicator four under the delivery of surgical care [1]. It was then considered by the WHO as a good gross indicator for access to surgery [2]. Post-operative mortality was estimated and ranked to be the third highest cause of death when compared with the global burden of disease report [3]. Following ischemic heart disease and stroke, post-operative death was estimated to be 7.7% of deaths globally [3]. This raises concerns if surgery is safe and affordable, especially in the low-resource setting. An approximate 1.4 million deaths could be prevented annually in LMICs. These avertable deaths further contribute to the overall development of a country.

According to the World Bank, India is home to a 1.42 billion population, with a gross domestic product per capita of $2,484 in 2023, classifying India as an LMIC. India also has a surgical specialist workforce of surgeons, anaesthetists, and obstetricians (SAO) of seven per 100,000 population, which is below the 2030 interim recommended of 20 SAO workforce according to LCoGS [1,4]. The POMR will provide a general understanding of the delivery of surgical care from the surgical workforce, surgical volume, and surgical safety [2]. POMR studies were scarcely documented in a non-government organisation (NGO) setting in India. Thus, we aimed to assess the POMR and perform a situational analysis with a surgical assessment tool (SAT) of a low-resource NGO hospital in India. 

This article was previously presented as a meeting abstract at the 82nd International College of Surgeons United States Section meeting on the 8th of July 2021. This poster won a scientific award at the meeting under the global surgery category.

## Materials and methods

Vivekananda Memorial Hospital (VMH) is a 100-bed rural-tribal hospital, run by a local NGO Swami Vivekananda Youth Movement. The hospital is a multi-specialty hospital that serves approximately 300,000 population [5]. The hospital has three operating theatres, three recovery beds, one blood storage unit, one radiographic machine, one general surgeon, one orthopaedic surgeon, four obstetricians, and one paediatrician who task shifts as an anaesthetist. The hospital has a constant supply of clean water and stable electricity with a generator as a backup. Other than providing inpatient care, VMH provides mobile clinics with a makeshift ambulance to follow up with patients’ conditions, postoperative outcomes, and perinatal care.

The perioperative mortality rate is defined as death following surgery and anaesthesia within two time periods: on the day of surgery, including intraoperative death, and before discharge from the hospital or within 30 days of surgery, whichever is sooner [1]. This definition was conservatively adopted as the majority of LMICs have difficulty following up with patients in rural areas. Procedures were defined as the incision, excision, or manipulation of tissue that needs regional or general anaesthesia or profound sedation to control pain. Surgical disciplines from general surgery, orthopaedics, obstetrics and gynaecology (O&G), otolaryngology, and ophthalmology procedures were all included in the study. Physical records are only kept for up to five years in VMH. ​​​​​​Thus, data from 01 January 2016 until 29 February 2020 were collected retrospectively using the SAT for the entire month of February 2020.

Inclusion and exclusion criteria

We included all patients who have undergone a procedure at VMH, regardless of the department for the surgical volume from 01 January 2016 until 29 February 2020. Mortality data were included if they went for an operation. Patients who were discharged against medical advice (DAMA) and mortality during transfers to other hospitals were also included. We excluded investigation procedures such as colonoscopy and oesophago-gastro-duodenoscopy.

Surgical volume data acquisition

We first identified the surgical volume by extracting the total surgery done from the operation list registry. The data extracted from the registry were the surgical department, surgical urgency, and the surgical procedures performed. All operative surgery done is routinely entered into the registry book.

Mortality data acquisition

The perioperative deaths were initially screened through with the surgeons to get a general number of deaths. Data on perioperative mortality were collected from the records unit. These mortalities were validated by the operating surgeon. Patients who DAMA were also tracked to screen for potential patients who could have died postoperatively during the recovery periods at home. Death during transfers to other hospitals was also screened for potential cases who needed emergency surgery but died during the transfer. Before the analysis of mortality, the data were further cross-checked with the Morbidity and Mortality Committee and documentation to validate the mortality. These data were managed by the administrative and records-keeping department that operated on softcopies and hardcopies of documentation. These data were collected by going through the physical files and were uploaded and stored into encrypted cloud storage. All patient names, identification, and registration numbers were deidentified.

Data analysis

The patient data were then peer-reviewed among the authors and descriptively analysed with Microsoft Excel (Microsoft® Corp., Redmond, WA), and risk analysis was done with MedCalc [6]. The analysed data fields were sent back to the hospital management to further understand their mortality data digitally to learn and provide better services to the community.

A modified SAT was used to assess the preparedness of surgical services in VMH in terms of infrastructure, service delivery, workforce, information management, and financing [7].

Ethics approval

Ethics was approved by the Swami Vivekananda Youth Movement Institutional Review Board. IRB (Health) No: 07/2019-20, approved on 22 February 2020.

## Results

Between January 2016 and February 2020, a total of 1,860 surgeries were performed across the departments of General Surgery, Orthopaedics, and O&G. Of these, 1,472 (79.1%) elective surgeries and 388 (20.9%) emergency surgeries were performed. General Surgery accounted for 614 (33%) procedures, with 561 (91.4%) being elective and 53 (8.6%) emergency. The Orthopaedics department performed 200 (10.8%) procedures, 192 (96%) were elective, and eight (4%) were emergency surgeries. The O&G department performed 1,046 (56.2%) procedures, of which 719 (68.7%) were elective and 327 (31.3%) emergency (Table 1).

**Table 1:** Operations performed in Vivekananda Memorial Hospital (VMH) Ear Nose Throat (ENT), Lower-Segment Caesarean Section (LSCS)

Department n (%)	Urgency (%)	Procedures performed	Number of procedures n(%)
General Surgery, 614 (33%)	Emergency, (8.6%)	Acute appendicitis	37 (6.0%)
		Open laparotomy for peritonitis	9 (1.5%)
		Necrotising soft tissue infection	7 (1.1%)
	Electives (91.4%)	Small incision cataract surgery	111 (18.1%)
		Fissure in Ano repair	84 (13.7%)
		Hernia repair	64 (10.4%)
		ENT	45 (7.3%)
		Circumcision	38 (6.2%)
		Hemorrhoidectomy	36 (5.8%)
		Laparoscopic cholecystectomy	33 (5.4%)
		Lumpectomy	25 (4.1%)
		Incision and drainage	17 (2.8%)
		Varicose veins stripping	17 (2.8%)
		Pterygium excision	15 (2.4%)
		Thyroidectomy	14 (2.3%)
		Breast abscess	13 (2.1%)
		Others	49 (8.0%)
Orthopaedics, 200 (10.8%)	Emergency (4%)	Necrotising fasciitis	5 (2.5%)
		Gangrene	3 (1.5%)
	Elective (96%)	Fracture fixation	125 (62.5%)
		Wound debridement	26 (13%)
		Amputation	13 (6.5%)
		Implant removal	12 (6%)
		Others	16 (8%)
Obstetrics and Gynaecology, 1046 (56.2%)	Emergency, (31.3%)	Emergency LSCS	315 (30.1%)
		Manual removal of the placenta	5 (0.5%)
		Laparotomy	3 (0.3%)
		Removal of ectopic pregnancy	3 (0.3%)
		Ovarian detorsion	1 (0.1%)
	Elective (68.7%)	Laparoscopic tubal occlusion	210 (20.1%)
		Elective LSCS	117 (11.2%)
		Abdominal hysterectomy	101 (9.7%)
		Vaginal hysterectomy	71 (6.8%)
		Dilatation and curretage	28 (2.7%)
		Tubectomy	14 (1.3%)
		Medical termination of pregnancy	12 (1.1%)
		Laparoscopic abdominal vaginal hysterectomy	12 (1.1%)
		Cervical cerclage	11 (1.1%)
		Others	33 (3.1%)

**Figure 1:**
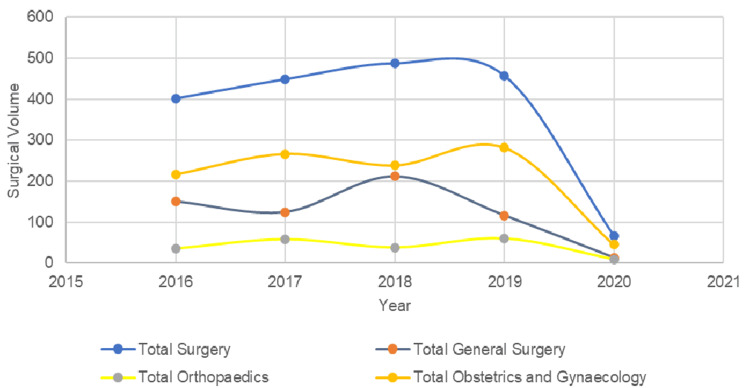
Yearly surgical volume trend performed at Vivekananda Memorial Hospital (VMH)

After adjustment, the overall surgical volume averaged 448.25 (95% CI: 391.52-504.96) procedures per year, which translates to 112 (95% CI: 130.52-168.32) procedures per 100,000 population annually. There were 92.25 (95% CI: 68.21-116.29) emergency procedures performed per year after adjustment. Departmentally, the mean annual surgical volume was 150.25 (95% CI: 81.79-218.71) for General Surgery, 47.75 (95% CI: 26.95-68.55) for Orthopaedics, and 204.16 (95% CI: 112.01-296.34) for O&G (Figure 1).

There were three perioperative deaths (0.16%) recorded, two were in General Surgery and one in O&G. The relative risk (RR) of perioperative mortality for emergency cases compared to elective cases was 1.9 (95% CI: 0.17-20.87) overall. The RR of perioperative mortality under General Surgery is 1.9 (95% CI 0.17-20.89), and O&G had an RR of 1.2 (95% CI: 0.45-29.37).

Situational analysis

Under infrastructure, although the electricity gets cut off sometimes, the available generators provide backup when needed for surgery. Running water is always available, but they required to be treated within the hospital before use. Internet and oxygen supply are almost always available.

Relevant data collected from the visiting mobile clinics around the area are uploaded to a cloud where VMH stores clinical data during clinic visits. Oxygen supply can run out similar to any hospital because of the limited tank reserve, but this is refilled on demand weekly. The radiographic tools are limited to X-rays and ultrasound in VMH; however, patients who can afford computed tomography (CT) scans and magnetic resonance imaging (MRI) usually get them done in another hospital that provides such services and is treated in VMH. Depending on the planned surgeries, blood products are ordered from a nearby blood bank, within two hours of reach, and are stored in the blood storage unit. VMH has a fully functional laboratory blood work allowing preoperative and postoperative monitoring in cases when patients are required to be referred to tertiary care (Table 2). In terms of service delivery, this hospital can provide all bellwether procedures, except for open fracture fixation where implants are concerned. Comparing the service to the Disease Control Priorities 3rd edition (DCP3) on essential surgery, VMH provides 32 procedures out of 44 procedures, which includes procedures to be provided in both primary and tertiary centres. Within the first-level hospital’s essential procedures enlisted, 20 out of 26 procedures are provided at VMH. Only procedures related to trauma and repair of bowel perforations were not performed (Table 3) [8].

**Table 2:** Modified surgical assessment tool to assess the preparedness of surgical services in VMH in terms of infrastructure, service delivery, workforce, information management, and financing Intensive Care Unit (ICU), Intravenous (IV), Computed Tomography (CT), Magnetic Resonance Imaging (MRI), White Blood Count (WBC), Blood Urea Nitrogen (BUN), Sodium (Na), Potassium (K), Prothrombin Time (PT), Partial Thromboplastin Time (PTT), International Normalised Ratio (INR), Human Immunodeficiency Virus (HIV), Obstetrics and Gynaecology (OBGYNs), Caesarean sections (C-sections), Laryngeal Mask Airway (LMA), Deep Vein Thrombosis (DVT) Source: Ref [7]

Modified surgical assessment		
General Infrastructure – How often is this item available and functional?		
Electricity/operational power generator	76-99% (Almost always)	
Running water	100% (Always)	
Internet	76-99% (Almost always)	
Oxygen	76-99% (Almost always)	
Total number of inpatient hospital beds	100 (Always)	
Total number of surgical beds	100 (Always)	
Total number of functioning operating rooms (major and minor)	3	
Total number of post-anaesthesia care beds (0 for none)	3	
Total number of advanced care/ICU beds	3	
Total number of functional ventilators in the ICU	3	
Pharmacy – How often is this available for surgery?		
Inhalational general anaesthesia	100% (Always)	
IV sedation anaesthesia (Ketamine, Midazolam, Propofol)	100% (Always)	
Spinal anaesthesia	100% (Always)	
Regional anaesthesia available	100% (Always)	
Peri-operative antibiotics	100% (Always)	
IV fluids	100% (Always)	
Muscle relaxants/paralytics	100% (Always)	
Sedatives	100% (Always)	
Vasopressors	100% (Always)	
Post-op narcotics	100% (Always)	
Radiology – How often do you have access to functioning radiology equipment?		
X-ray machine	100% (Always)	
Ultrasound	100% (Always)	
CT scanner	0 (Never)	
MRI scanner	0 (Never)	
Blood Supply		
How often are you able to administer a blood transfusion within 2 hours in your facility?	76-99% (Almost always)	
Laboratory		
How often is the lab able to run a Complete Blood Count (haemoglobin, haematocrit, WBC, platelets)?	100% (Always)	
How often is the lab able to run a chemistry panel (BUN, creatinine, Na, K, etc.)?	100% (Always)	
How often is the lab able to run coagulation studies (PT, PTT, INR)?	100% (Always)	
How often is the lab able to do a urinalysis?	100% (Always)	
How often are you able to screen for an infectious panel (HIV, hepatitis virus)?	100% (Always)	
Access and Referral Systems		
What is the population served by this facility?	300,000	
What percentage of your patients can reach the hospital within 2 hours of travel?	76-99% (Almost always)	
Total number of patients that you refer for surgical intervention to a higher level facility per year	N/A	
Surgeon / Anaesthesiologist / Obstetrician Workforce		
Providers	Full time	Part-time
What is the number of certified surgeons who work at this hospital (full vs part-time)	4	3
Number of certified paediatric surgeons	0	0
Number of certified OBGYNs	1	3
Number of certified anaesthesiologists	0	0
Number of general doctors providing surgery	1	0
Number of general doctors providing C-sections	1	0
Number of general doctors providing anaesthesia	1	0
Number of non-physicians providing surgery	0	0
Number of non-physicians providing C-sections	0	0
Number of non-physicians providing anaesthesia	0	0
Number of midwives	2	0
Number of nurses on the surgical wards	4	0
Number of certified radiologists	0	0
Number of certified pathologists	0	0
Number of certified pharmacists	0	0
Number of certified biomedical technicians	0	0
How often is a surgical provider available 24 hours a day?	51-75% (Often)	
How often is an obstetrics/gynaecology provider available 24 hours a day?	100% (Always)	
How often is an anaesthesia provider available 24 hours a day?	100% (Always)	
Continuing Medical Education		
How often do you offer continuing medical education to your staff each year?	Monthly	
Operating Room Equipment and Supplies – How often are the following equipment available and functional for surgery?		
Total number of functional anaesthesia machines in the Ors	3	
Pulse oximetry	5	
Adult oropharyngeal airway	100% (Always)	
Paediatric oropharyngeal airway	100% (Always)	
Adult endotracheal tube	100% (Always)	
Paediatric endotracheal tube	100% (Always)	
Adult laryngoscope	100% (Always)	
Paediatric laryngoscope	100% (Always)	
Adult facemask bag valve	100% (Always)	
Paediatric facemask bag valve	100% (Always)	
Difficult airway kit (LMA)	100% (Always)	
Adult Magill forceps	100% (Always)	
Paediatric Magill forceps	100% (Always)	
Blood pressure monitor or cuff	100% (Always)	
Pulse oximeter	100% (Always)	
Stethoscope	100% (Always)	
Suction apparatus	100% (Always)	
Thermometer	100% (Always)	
Nasogastric Tube	100% (Always)	
Light source	100% (Always)	
Chest tube	100% (Always)	
Electrocautery	100% (Always)	
Autoclave/Sterilizer	100% (Always)	
Forceps	100% (Always)	
Syringes with needles	100% (Always)	
Scalpel	100% (Always)	
Scissors	100% (Always)	
Needle holder	100% (Always)	
Retractor	100% (Always)	
Sterile gloves	100% (Always)	
Urinary catheters	100% (Always)	
Tourniquet	100% (Always)	
Face masks	100% (Always)	
Gowns	100% (Always)	
Disinfectant hand wash	100% (Always)	
Sterilizing skin prep	100% (Always)	
Eye protection	100% (Always)	
Sharps disposal container	100% (Always)	
Non-sterile Examination Gloves	100% (Always)	
Sutures	100% (Always)	
Information Management System		
What is the method of record keeping in your hospital?	Both written and electronic	
Are there personnel in charge of maintaining medical records?	Yes	
Are charts accessible across multiple visits for the same patient?	Yes	
How often is data prospectively collected for patient outcomes, such as surgical site infection, post op stroke, DVT, etc.?	100% (Always)	
How often is data prospectively collected for post-operative mortality rate?	1-25% (Rarely)	
How often are you required to report information to the Ministry of Health or an equivalent agency?	Never	
Do you use telemedicine?	No	
Research		
How often does the hospital participate in quality improvement projects, such as mortality & morbidity conferences?	Yearly	
How many ongoing research projects does the hospital have?	5	
How many ongoing research projects does the Department of Surgery have?	3	
Health Financing and Accounting		
What percentage of your patients have health insurance?	51-75% (Heavily subsidized in comparison to other private hospitals) If patients are part of a tribal(indigenous) group, they are given free medical care. Non-tribal groups will still need to pay about 50% of admission fee.	

**Table 3:** The list of essential procedures according to Disease Control Priorities 3rd edition (DCP3) † - Procedures performed in VMH. Visual impairment surgeries were performed by a visiting ophthalmologist. Source: Ref [8]

Types of procedures	Community and primary health centre	First-level hospital	Second and third-level hospitals
Dental	Extraction		
	Drainage of dental abscess		
	Treatment for caries		
Obstetrics and Gynaecology	†Normal delivery	†Caesarean birth	Obstetric fistula repair
		†Vacuum extraction/forceps delivery	
		†Ectopic pregnancy	
		†Manual vacuum aspiration and dilation and curettage	
		†Tubal ligation	
		†Vasectomy	
		†Hysterectomy for uterine rupture or intractable postpartum haemorrhage	
		†Visual inspection with acetic acid and cryotherapy for precancerous cervical lesions	
General Surgery	†Drainage of superficial abscess	†Relief of urinary obstruction: catheterisation or suprapubic cystostomy	
	†Male circumcision	†Appendectomy	
		†Bowel obstruction	
		†Colostomy	
		†Gallbladder disease, including emergency surgery	
		†Hernia repair	
		†Hydrocelectomy	
		Repair of perforations	
Injury	†Resuscitation with basic life support measures	†Skin grafting	
	†Suturing laceration	†Tube thoracostomy	
	†Management of non-displaced fractures	†Escharotomy/fasciotomy	
		†Fracture reduction	
		†Irrigation and debridement of open fractures	
		Placement of external fixators and use of traction	
		Trauma laparotomy	
		Traumatic amputations	
		Resuscitation with advanced life support measures, including surgical airway	
		Burr hole	
Congenital			†Repair of anorectal malformations and †Hirshsprung disease
			†Repair of clubfoot
			Shunt for hydrocephalus
			Repair of cleft lip and palate
Visual impairment			†Cataract extraction and insertion of intraocular lens
			†Eyelid surgery for trachoma
Nontrauma orthopaedic		†Drainage of septic arthritis	
		†Debridement of osteomyelitis	

The surgical workforce in VMH has seven surgeons, which equates to 1.75 SAO per 100,000 population. The information management system keeps both electronic and written records. There are staff in charge of maintaining these medical records, and patient charts are always accessible across multiple visits for the same patient. Data on patient outcomes are always prospectively collected in terms of morbidity but rarely collected on mortality. These data are tracked within the institution but are not required to be reported to the local authorities.

## Discussion

Globally, NGOs do not routinely report their surgical volume and perioperative mortality, but most NGOs do track them internally [9]. Following the narrative of LCoGS, partnering with NGOs to report LCoGS indicators became imperative to understand the surgical services provided to the population at a global scale [10]. To further understand how these NGOs provide surgical services, a survey on clinical, data collection, and health systems development was sent to 99 US-based international surgical and anaesthesia organisations. Forty-six of them responded to the survey, and two-thirds of the organisation performed below 500 procedures per year [9]. The surgical volume for this site provided 448 procedures per year, which is impressive considering the previous study only focussed on US-based NGOs. When compared with other NGOs around the world, 112 procedures per 100,000 population per year is comparatively higher than other NGOs that reported their surgical volume [11,12]. The case mix was variable depending on the NGOs, some concentrated their services on disease-specific procedures for cataracts or cleft lip and some, like VMH, provided a wide range of surgical procedures [9,12,13]. In terms of comparing to low-resource permanent hospitals, institutions in rural parts of Ethiopia had a similar infrastructure and surgical output. Their study included 10 hospitals where their hospital beds ranged from 20 to 350 beds, two for four operating rooms per hospital, and one to eight surgeons in each hospital. The median annual surgical procedures in these hospitals were 123 (56-421) per 100,000 population [14]. Surgical volume is reliant on many factors; however, similar infrastructure and number of surgical providers appear to be associated with comparable outputs in low-resource settings and among other NGOs globally [12,14].

POMR has been reported to be impressively low in many of these NGOs [10]. NGOs, such as Medecins Sans Frontieres (MSF), reported a range between 0 and 1%. Under MSF, they reported patients with an American Society of Anesthesiologist score (ASA) of 3-5, patients involved in violent injuries and conflict, and those who required emergency surgeries were associated with perioperative mortality [12]. POMR in VMH was 0.16% (three deaths), which is comparatively similar to MSF. There were only 388 (21%) emergency cases performed in VMH, which is generally much lower than the rest of the world, varying from 42% to 65% [15-17]. It is well-documented that emergency cases are more at risk of mortality than elective cases, and this is one of the reasons why the POMR in VMH is low where emergency cases are generally lower than other NGOs that reported their surgical output [12,15]. Considering studies reporting intraoperative mortality as high as 5-10% [12], the surgical services in this site are considered safe. Although POMR provides a good general indicator for surgical safety, it should be used with caution. Perioperative mortality is multifaceted, where mortality could be affected by preoperative, operative, and postoperative factors [18]. These factors have multiple stages where clinical decisions are made; thus, having knowledgeable and experienced surgical providers was a major factor that contributed to the low POMR rate in VMH. This low mortality from NGOs may also be related to the careful case selection criteria, where surgery is not provided to patients who are deemed high risk, given the limited capacity of many NGOs [11,12]. Understanding the timing of referral and the limitations of an institution at a district level is crucial. This can help decide whether to further escalate to a tertiary centre for better care in both preoperative and postoperative stages. Most of the trauma cases in LMICs die before reaching a district hospital, which could also be the case for VMH of lower emergency cases being operated on [8].

Understanding the strength of the surgical system will help in analysing surgical volume and POMR at these NGO sites in terms of infrastructure, service delivery, workforce, information management, and financing [19]. This situational analysis was done with the SAT developed by WHO and the Program of Global Surgery and Social Change group [7,19]. In the present study, we collected data with a modified SAT and the types of surgical services that VMH provides according to DCP3 [8]. From the situational analysis (Table 2), VMH is a very well-prepared hospital to provide both exploratory laparotomy and caesarean delivery from the bellwether procedures, along with the majority of the first-level care procedures and some third-level hospital care procedures for those who need them [8]. External fixators and implants are not performed due to the lack of funding for implants and their maintenance. Most of our patients do not return to the hospital for further follow-ups, and this would result in a loss of equipment and an increased risk of infection even though external fixators could be safely removed and reused after sterilisation [20-22]. Implants generally are also expensive, which is the reason why VMH does not provide those types of services. Since most open fractures are not immediately life-threatening but urgent, VMH refers these types of cases as soon as possible to tertiary centres after stabilisation.

Trauma care is expensive because of the equipment, maintenance, and manpower to operate and interpret them and the electricity needed for diagnostics, such as CT scans and MRIs. A systematic review of the cost of trauma care in LMICs varied from USD$14 to USD$17,400 [23], which is still considered expensive as the cost range is wide and the higher range is up to USD$17,400. These machines were deemed to have a better cost-to-utility ratio in higher centres where complicated non-trauma cases, trauma centres, and higher case volume can make the best out of them with a radiologist, instead of only treating trauma patients in VMH. In terms of financing, VMH relies on social capital and donations to provide healthcare. Although much of the equipment and materials are limited, quality and affordable healthcare is still provided to their population. Even though the human resources of the surgical ecosystem are lacking, VMH fulfils most of the ideal infrastructure and equipment required (Table 4).

**Table 4:** The Ideal 100-bed district (second-level hospital) according to Disease Control Priorities 3rd edition (DCP3) ¶ - The ideal hospital criteria fulfilled by Vivekananda Memorial Hospital (VMH).

Category requirement	100-bed district (second-level) hospital
Infrastructure	Inpatient facility of 100 beds, including several wards and an isolation ward
	¶Outpatient facility including an emergency room; operating rooms (at least two: one clean, one contaminated)
	¶Labor and delivery rooms
	¶Recovery room or intensive care unit
	Blood bank
	¶Pharmacy
	¶Clinical laboratory
	¶Radiology and ultrasonography suite
Equipment and supplies	¶Anesthetic machines and inhalation gases
	¶Monitors (electrocardiogram, blood pressure, pulse oximetry)
	¶Fully equipped operating room
	¶Fully equipped delivery room
	¶Fully equipped recovery room or intensive care unit
	¶Respirators and oxygen supply
	¶Blood products and intravenous fluids
	¶Basic microbiology equipment
	¶Pharmaceuticals, (anesthetics, analgesics, antibiotics)
	¶Surgical materials (drapes, gowns, dressings, gloves), and other consumables (disposable equipment and devices)
Human Resource	Nurses (50+)
	Midwives (5+)
	Anesthetists (2–3)
	Anesthesiologist (1)
	Primary care physicians (4)
	¶Obstetrician/gynecologist (1 or 2)
	General surgeons (2)
	Pharmacy assistants (2)
	Pharmacist (1)b
	¶Radiology technician (1)
	Radiologist (1)
	Physiotherapist (1)

Remarkably this hospital can still demonstrate an international standard of POMR even with a lack of staffing, which was also similarly demonstrated by other institutions with low-resource settings [12,19].

Recommendation

Digitising documentation and storing patient information will benefit hospitals in these settings. This could generate a better analysis of POMR and mortality meetings in the future and provide site-specific feedback audits to further reduce mortality. A hybrid model of documentation can be considered, and the use of manual documentation should then be uploaded to cloud storage where digital data could be accessed. During electricity cuts, digital files may be difficult to access; therefore, having a system where all local computers are synced appropriately could also provide some form of security in terms of electricity, data management, and data retrieval.

Inspiring more doctors to work in a rural setting and providing clinical exposure to graduating medical students can help. This can increase the workforce on the ground to provide different experiences, which is going to be helpful in many hospital settings. Having incentives such as free child education, accommodation, and better pay from investors or governments might be able to attract specialists to work in these rural areas.

Limitations

All medical records were written manually, and some clinical data were either missing, illegible, or not documented. The sample size for mortality studies is usually difficult to analyse in these settings because of the small mortality data.

## Conclusions

In a low-resource NGO setting, POMR can meet international standards, reaching as low as 0.16%. VMH is a good example where access to safe surgical care is possible in a low-resource setting. Emergency surgeries have twice the risk of mortality in comparison to elective cases here. Experienced specialists and careful patient selection are crucial in knowing when to transfer patients to higher centres. This can further reduce the rates of POMR in district hospitals and provide access to appropriate care in tertiary centres. This study highlights that high-quality surgical care can be effectively delivered even to the poorest of the population.

## Appendices

**Table 5:** Procedures categorised under "Others" from Table 1

Procedures	
General Surgery	Count (n)
Hydrocele	8
Excision of Pilonidal sinus	6
Mastectomy	6
Tissue Excision	5
Orchidopexy	4
Removal of foreign body	3
Dilatation of anorectum	3
Varicocelectomy	3
Excision biopsy	2
Urachal cystectomy	2
Parotidectomy	2
Open Roux en Y	1
Excision of polydactyly	1
Omphalitis	1
Electrocautery	1
Cystectomy	1
Orthopaedics	
Tenotomy	5
Split skin graft	4
Laminectomy	2
Tendon repair	2
Contracture release	1
Limb lengthening	1
Compartment syndrome	1
Obstetrics and Gynaecology	
Polypectomy	8
Cystectomy	7
Copper T removal	5
Pelvic floor repair	4
Lower segment caesarean section surgical site infection	2
Bartholin cystectomy	2
Recanalization	2
Endometrial scar excision	1
Cervical dilatation	1
Ovarian drilling	1
